# Structural Characterization of Nanobodies during Germline Maturation

**DOI:** 10.3390/biom13020380

**Published:** 2023-02-17

**Authors:** Clarissa A. Seidler, Janik Kokot, Monica L. Fernández-Quintero, Klaus R. Liedl

**Affiliations:** Department of General, Inorganic and Theoretical Chemistry, Center for Molecular Biosciences Innsbruck (CMBI), University of Innsbruck, 6020 Innsbruck, Austria

**Keywords:** camelid V_H_H antibodies, affinity maturation, molecular dynamics, enhanced sampling, Markov-state model

## Abstract

Camelid heavy-chain antibody variable domains (V_H_H), nanobodies, are the smallest-known functional antibody fragments with high therapeutic potential. In this study, we investigate a V_H_H binding to hen egg-white lysozyme (HEL). We structurally and dynamically characterized the conformational diversity of four V_H_H variants to elucidate the antigen-binding process. For two of these antibodies, not only are the dissociation constants known, but also the experimentally determined crystal structures of the V_H_H in complex with HEL are available. We performed well-tempered metadynamics simulations in combination with molecular dynamics simulations to capture a broad conformational space and to reconstruct the thermodynamics and kinetics of conformational transitions in the antigen-binding site, the paratope. By kinetically characterizing the loop movements of the paratope, we found that, with an increase in affinity, the state populations shift towards the binding competent conformation. The contacts contributing to antigen binding, and those who contribute to the overall stability, show a clear trend towards less variable but more intense contacts. Additionally, these investigated nanobodies clearly follow the conformational selection paradigm, as the binding competent conformation pre-exists within the structural ensembles without the presence of the antigen.

## 1. Introduction

*Camelidae*, similar to cartilaginous fish, are equipped with heavy-chain-only antibodies (HCAbs). The variable domains of antibodies are also referred to as nanobodies or single-domain V_H_H. [1,2,3]. In contrast to *Elasmobranches*, where the HCAbs diversified at least 220 million years ago, the development of camelid HCAbs is much more recent [4].

**Figure 1:**
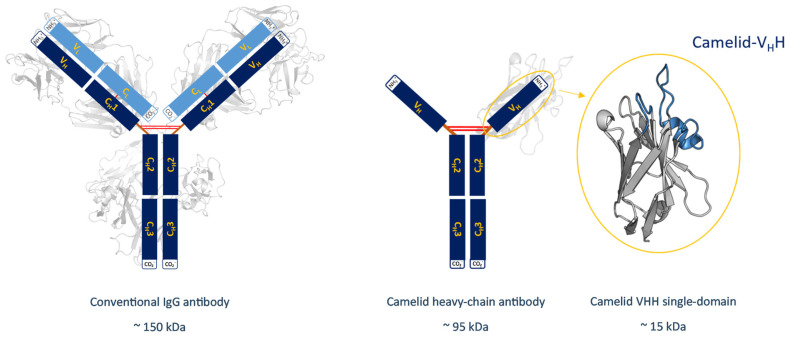
Overview showing the different sizes of antibodies and their derivates. A structural comparison between a conventional IgG-type antibody and a camelid heavy chain antibody with its variable fragment is shown. Next to the schematic representation of the V_H_H, the structure of the respective single domain is depicted and the CDR 3 and the CDR1 loops highlighted in blue. Additionally, the molecular weights of the structures are shown, which range from 150 kDa for the conventional IgG-type antibody, to only 15 kDa for the V_H_H single domain.

**Figure 2:**
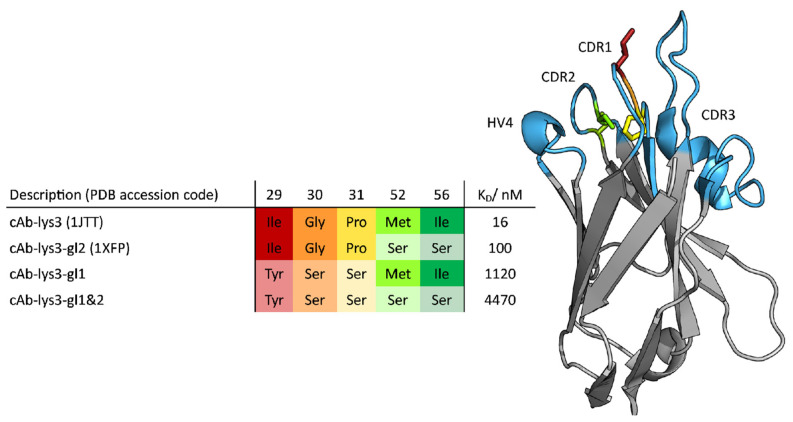
Tabular overview of the introduced point mutations (**left**). The introduced point mutations and respective positions are color-coded and shown as sticks for the cAB-lys3-gl2 (PDB: 1XFP) (**right**). The CDR loop regions CDR1, CDR2, CDR3 and the hypervariable region HV4 are highlighted in light blue and labeled.

Structurally, camelid HCAbs consist of only two heavy chains, which can be subdivided into two constant domains and one variable domain. Nanobodies lack the hydrophobic interface, which is usually found in IgG-type antibodies. Nevertheless, they are functional without their light-chain counterparts due to the introduction of five, mainly hydrophilic, residues [5]. These substitutions, namely Leu11Ser, Val37Phe; Tyr, Gly44Glu, Leu45Arg; or Cys, Trp47Gly (Kabat nomenclature), are conserved among all nanobodies and are located at the surface of the variable domain [6,7]. The introduced amino acids minimize the risk of aggregation, which is one of several reasons why nanobodies are a valuable alternative to conventional antibodies as pharmaceuticals [3,8,9,10,11]. A structural comparison between a conventional IgG-type antibody and a camelid antibody with its variable domain is illustrated in Figure 1. The missing first constant domain, referred to as C_H_1, and the missing light chains cause a reduction in the molecular weight, as shown in this figure. The upper tip of the domain has a weight of about 15 kDa and represents the binding site of a camelid V_H_H. This part also known as the paratope, consists of three complementarity determining regions (CDR) named CDR 1, CDR 2 and CDR 3, which are illustrated in light blue in Figure 2, together with the hypervariable region 4 (HV4). In conventional antibodies, the CDR 3 loop is located in a central position of the binding interface and is known to be predominantly responsible for polypeptide recognition [12]. This loop has been widely studied and discussed in multiple papers and is known to play a significant role in antigen binding. Its promiscuity has been linked to its flexibility, although the correlation between specificity and flexibility seems to be only one of several mechanisms that contribute to an increased binding affinity [13,14,15].

Recent studies suggest alternative mechanisms for an increase in affinity. Jeliazkov et al. focused on the CDR-H3 loop of antibodies, and observed that affinity maturation does not necessarily result in a decrease in flexibility [13]. Nevertheless, the CDR-H3 loop was shown to be particularly flexible compared to other paratope regions, and its diversity was suggested to be one responsible characteristic for antibodies’ promiscuity [14]. In antibodies belonging to the V_H_H class, an even higher importance is attributed to the CDR 3 loop as it is particularly long and allows the detection of buried binding sites on the antigen (epitope) [16,17]. The shape complementarity of this loop to the structure of the epitope allows binding with similar affinity compared to much larger antibodies having more extended paratope regions [18,19]. In the present case, the Camelid V_H_H domain is captured in complex with the hen egg-white lysozyme (HEL), a bacteriolytic enzyme. The HEL has been intensively studied and numerous crystal structures are available, making it a good model system [20,21].

In this paper, the influence of point mutations on conformational diversity was characterized for a dataset of camelid V_H_H domains with different binding affinity [22,23,24]. A total of four sequences with respective binding affinities were available differing in up to five mutations within the CDR 1 and CDR 2 loops.

This work aims to investigate mechanisms of antigen recognition through a kinetic and thermodynamic description of the conformational diversity of the antigen binding loops. Therefore, conventional molecular dynamics simulations have been performed in combination with enhanced sampling techniques, in order to overcome limitations in the timescale, and for being able to reconstruct the free-energy surfaces. In particular, well-tempered metadynamics simulations turned out to be a well-suited sampling technique for these comparably small systems.

## 2. Materials and Methods

The investigated dataset consists of two experimentally determined crystal structures (PDB codes: 1JTT, 1XFP) and two sequences which were modeled using the crystal structures as templates. Each of the four variants were simulated with and without the antigen present, resulting in a total of eight starting structures for molecular dynamics simulations, namely cAb-gl1+2, cAb-gl1, cAb-gl2 and cAb as the unbound structures, and cAb-lys3-gl1+2, cAb-lys3-gl1, cAb-lys3-gl2 (1XFP) and cAb-lys3 (1JTT) as the structures in complex with the HEL.

All starting structures were carefully prepared by use of the MOE (Molecular Operating Environment, Chemical Computing Group, version 2020.09) Protonate3D tool [25,26]. Therefore, the C-terminal ends were capped with N-methylamine (NME) and the charges were neutralized by the use of a uniform background charge [27]. The proteins were then soaked in cubic water boxes of TIP3P water molecules with a minimum wall distance of 10 Å [28,29].

For all simulations, parameters of the AMBER force field 14SB were used [30]. Equilibrations were performed by adopting a well-established multi-step equilibration protocol [31].

All images were created by use of the PyMOL molecular graphics system, version 2.4.1 [32].

### 2.1. Metadynamics Simulations

In order to sample a broad conformational space, we performed metadynamics simulations to investigate the conformational diversity of all available single-domain variants. In particular, well-tempered metadynamics simulations were chosen, which allowed us to enhance the sampling on a particular pre-defined set of collective variables (CVs) [33,34,35,36,37].

All metadynamics simulations were performed in GROMACS by use of the PLUMED 2 implementation [38,39,40]. Since CDR loops 2 and 3 are the ones mainly involved in binding, we chose as CVs a linear combination of the sine and cosine of the ψ torsion angles of the residues of these two CDR loops. Those were computed using the MATHEVAL and the COMBINE implementations of PLUMED 2 [40]. The ψ angles are able to capture all crucial conformational movements comprehensively [40,41,42]. We used a Gaussian height of 10.0 kJ/mol and a width of 0.3 rad. The deposition occurred every 1000 steps, while a bias factor of 10 was used.

The simulations were performed at a temperature of 300 K in an NpT ensemble. The conservation of a constant temperature was achieved by use of a Langevin thermostat [43]. The atmospheric pressure was set by use of a Berendsen weak-coupled external bath [44]. Additionally, all bonds to hydrogen atoms were restrained by the use of the SHAKE algorithm, in order to allow a time step of 2 fs [45,46].

### 2.2. Molecular Dynamics Simulations

The obtained enhanced sampling simulation trajectories were clustered by the use of the CPPTRAJ implementation of the AMBER software package [47,48]. Therefore, a hierarchical average linkage approach was chosen, and it was clustered on the Cα-atoms of the three CDR loops using a distance cut-off criterion of 1.2 Å.

The resulting cluster representatives were then used as starting structures for conventional and unbiased molecular dynamics simulations (cMDs). Each starting structure was simulated for a total of 200 ns in an NpT ensemble at a temperature of 300 K. As for the metadynamics simulations, the Langevin thermostat and a Berendsen manostat were used and the bonds involving hydrogen atoms were restrained by the use of the SHAKE algorithm [43,44,45,46].

### 2.3. Analysis of the Simulation Trajectories

The local flexibility of the single residues during the cMD simulations was determined by calculating the root mean square fluctuations (RMSF). This was achieved by the use of AMBER’s CPPTRAJ implementation [47]. The structures of the antibody variable fragments without considering the antigens, were therefore aligned on all Cα-atoms of the crystal structure and the fluctuations calculated on the Cα-atoms in a mass-weighted manner [47].

The obtained simulation trajectories were analyzed with a principal component analysis (PCA) on the Cα-atoms of the CDR 2 loop, and of the binding residues of the CDR 3 loop, as those are the regions directly in contact with the antigen. For this analyses, the PyEMMA 2 python library was used. Additionally, the PCA spaces, using as input features the backbone torsions, and the Cα-atoms of all hypervariable loops individually, were investigated (Supplementary Figures S3–S5).

For the reduction in the dimensionality and the subsequent construction of a Markov State Model (MSM), a time-lagged independent component analysis (tICA) was performed again using the PyEMMA 2 python library. tICA was applied to identify the slowest degrees of freedom [49,50,51].

The obtained tICA space was therefore clustered geometrically by a k-means clustering algorithm in order to define a set of microstates [52]. For each simulation, a total number of 120 k-means clusters was defined. These microstates were then coarse-grained into macrostates by use of a fuzzy PCCA+ clustering algorithm, implemented in the used PyEMMA 2 library [49,53]. This way, kinetically relevant states were defined and transition probabilities between them could be calculated. To construct the Markov-state models we applied a lag-time of 15 ns and evaluated the reliability of the constructed MSM with the so-called Chapman–Kolmogorov test [54,55].

For the calculation of the contacts between antibody and antigen, as well as for the intermolecular contacts, the GetContacts tool provided by the University of Stanford was used (https://getcontacts.github.io/, accessed on 21 October 2022) [56]. This software is able to compute interactions based on pre-defined criteria. For the purpose of this study, the hydrogen bonds beneath a distance cut-off of 3.5 Å between all atoms were computed. Therefore the evolution of contacts for different stages of maturation could be quantified, and the contacts could be directly compared using a flare plot visualization.

## 3. Results

A well-established simulation protocol was applied to characterize the influence of point mutations on antigen recognition [31]. Therefore, the two crystal structures (PDB accession codes 1JTT and 1XFP) were simulated each for 1 μs with well-tempered metadynamics simulations. Based on the experimentally measured structures, additional point mutations were introduced according to the available sequence data, with the MOE modeling software package. The obtained structure models were simulated following the same protocol as the crystal structures.

For all four nanobody variants, experimentally determined dissociation constants were available. The investigated variants with their respective dissociation constants and point mutations are summarized in Figure 2.

The dataset is composed of a highly matured V_H_H variant (cAb-lys3, PDB accession code 1JTT) and three variants including mutations, which are required to obtain the germline antibody (cAb-lys3-gl1+2). Mutations were introduced in the CDR 2 loop (cAb-lys3-gl2, PDB accession code 1XFP), in the CDR 1 loop (cAb-lys3-gl1) and in both loops (cAb-lys3-gl1+2). As a direct result of the introduced mutations, the experimentally determined dissociation constants increase from about 16 nM up to 4470 nM. The available experimental data are summarized in Figure 2, where the mutations are illustrated structurally [22].

The CDR 3 loop consists of 24 residues and is stabilized by a disulfide bridge, joining a cysteine situated in the middle of the CDR 3 loop with a cysteine in the anchor region of the CDR 1 loop according to the Chothia enumeration scheme [57]. Additionally, a fourth hypervariable loop—the HV 4—is highlighted in Figure 2. This region is not directly involved in binding the antigen, but due to the strong structural correlations between the CDR loops, it can still influence the binding properties of the paratope, as was shown in the previous studies of T-cell receptors [58]. By analyzing the flexibility of individual residues using the Root Mean Square Fluctuation (RMSF) of the various variants, it was observed that the HV 4 loop becomes more rigid during the process of affinity maturation, as seen in the Supplementary Figure S1. Only for the cAb-lys3-gl2 variant a slightly higher flexibility compared to the most matured variant was found.

All variants were simulated with and without the hen egg-white lysozyme antigen present.

The resulting metadynamics trajectories were clustered on the Cα- atoms of the three CDR loops with the use of a hierarchical average linkage clustering algorithm, applying a distance cut-off criterion of 1.2 Å. Subsequently, the cluster representatives were simulated each for 200 ns. Thus, the overall sampling is dependent on the obtained number of clusters. The respective aggregated simulation times of the cMD trajectories are summarized in Table 1.

### Time-Lagged Independent Component Analysis (tICA) and Markov-State Models

**Figure 3:**
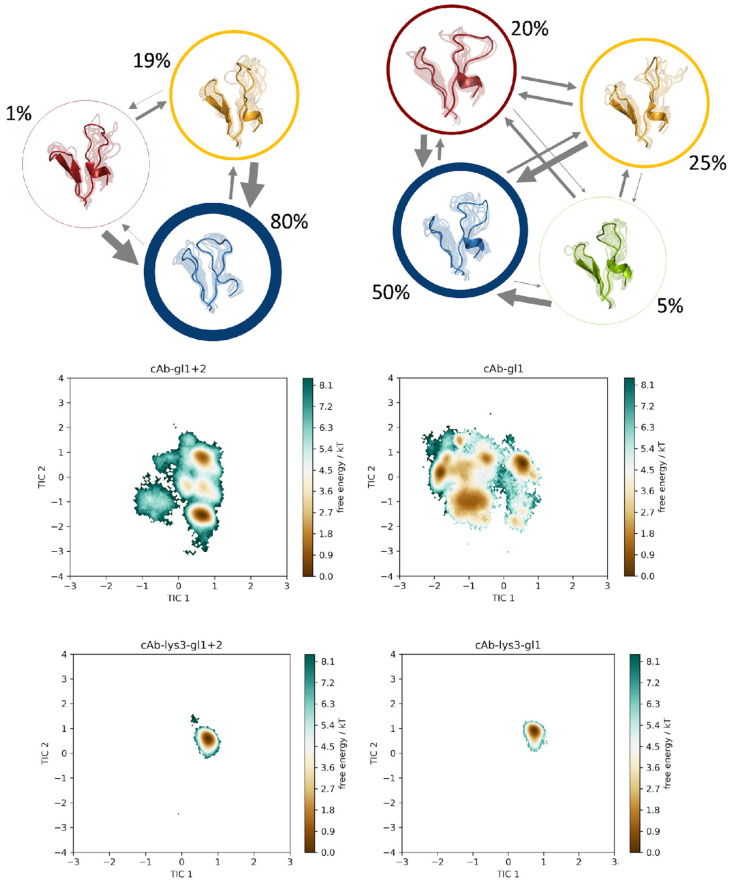
Markov-state models for the germline variant cAb-gl1+2 and for the cAb-gl1 variant. The simulations were performed without antigen present. The respective state probabilities are shown as percentages and reflected in the thickness of the respective circles. Faster transition timescales are represented as thicker arrows connecting the respective states. For each trajectory, the conformational tICA spaces are shown, projected into the same coordinate system together with the simulations of the bound structures (cAb-lys3-gl1+2 and cAb-lys3-gl1).

**Figure 4:**
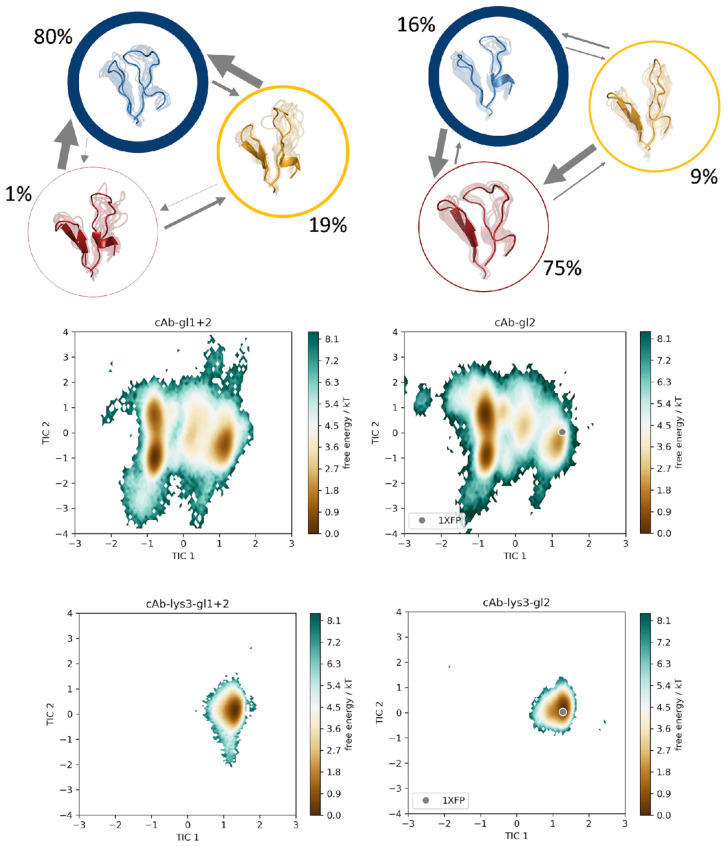
Markov-state models for the germline variant cAb-gl1+2 and for the cAb-gl2 variant. The simulations were performed without antigen present. The respective state probabilities are shown as percentages and reflected in the thickness of the respective circles. Faster transition timescales are represented as thicker arrows connecting the respective states. For each trajectory, the conformational tICA spaces are shown, projected into the same coordinate system together with the simulations of the bound structures (cAb-lys3-gl1+2 and cAb-lys3-gl2). The projection of the available crystal structure (PDB: 1XFP) is shown in the same coordinate system.

**Figure 5:**
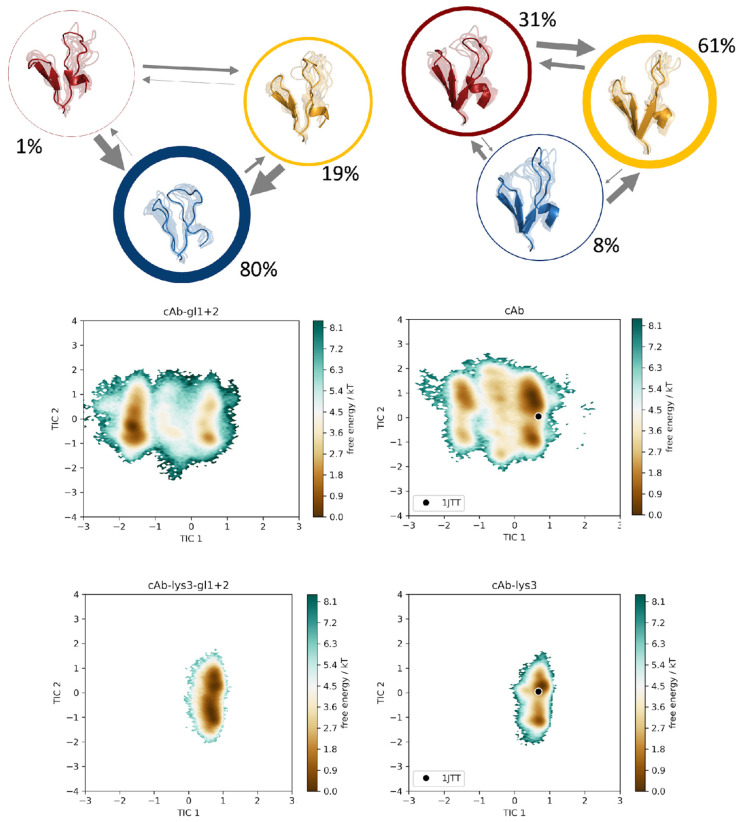
Markov-state models for the germline variant cAb-gl1+2 and for the cAb variant. The simulations were performed without antigen present. The respective state probabilities are shown as percentages and reflected in the thickness of the respective circles. Faster transition timescales are represented as thicker arrows connecting the respective states. For each trajectory, the conformational tICA spaces are shown, projected into the same coordinate system together with the simulations of the bound structures (cAb-lys3-gl1+2 and cAb-lys3). The projection of the available crystal structure (PDB: 1JTT) is shown in the same coordinate system.

We constructed a tICA, using the backbone torsions of the CDR 3 and CDR 2 loops as input features. In particular, of the CDR 3 loop, only the region directly involved in binding was considered (namely the sequence including residues D99-E108), since this loop is particularly long with a total of 24 residues and only this small fraction binds into the cleft of the antigen’s epitope. In Figure 3, the trajectories of the cAb-gl1+2 variant is compared to the cAb-gl1 variant, together with the two bound structures, namely cAb-lys3-gl1+2 and cAb-lys3-gl1 in the same tICA coordinate space. Analogously, this comparison was performed also for the cAb-gl1+2 variant with the cAb-gl2, with and without antigen present (Figure 4), and for the cAb-gl1+2 variant compared to the most matured variant, cAb, respectively, with and without antigen (Figure 5). The obtained tICA spaces were clustered by performing a k-means clustering, subdividing the trajectories in every 120 microstates. From the resulting microstates, a MSM was built for all unbound simulations individually, with the application of a lag time of 15 ns. The previously mentioned pairwise comparisons of the resulting macrostates with the affiliated combined tICA spaces are shown in Figure 3, Figure 4 and Figure 5 as well.

From the MSMs, we obtained the transition probabilities and kinetics between different minima in the solution. As previously mentioned, the germline variant with the lowest binding affinity was compared pairwise to each of the remaining trajectories. The tICA spaces, which were combined in one coordinate system together with the respective bound counterparts, showed no significant reduction in the sampled conformational space, but a clear change in the distributions of the Markov state probabilities was found.

For each of the trajectories, cAb-gl1+2, cAb-gl2 and cAb, a total of three macrostates was found, while an additional fourth state could be observed for the cAb-gl2 variant. Interestingly, the binding competent conformation was observed as the dominant state (61%) in the solution for the antibody with the highest affinity, namely the cAb variant, corresponding to the PDB entry 1JTT. For the cAb-gl1 structure, this conformation was observed with a state probability of only 25%, for the cAb-gl2 with only 9% and for the germline antibody, the binding competent conformation had a probability of 19%. This is particularly exciting because the binding competent conformation was present in the other variant as well, but with a much lower state probability compared to the most matured variant.

Therefore, we suggest a conformational selection binding mechanism for the V_H_H variants, as the structure capable of binding the antigen is pre-existing, even in absence of the antigen [59].

**Figure 6:**
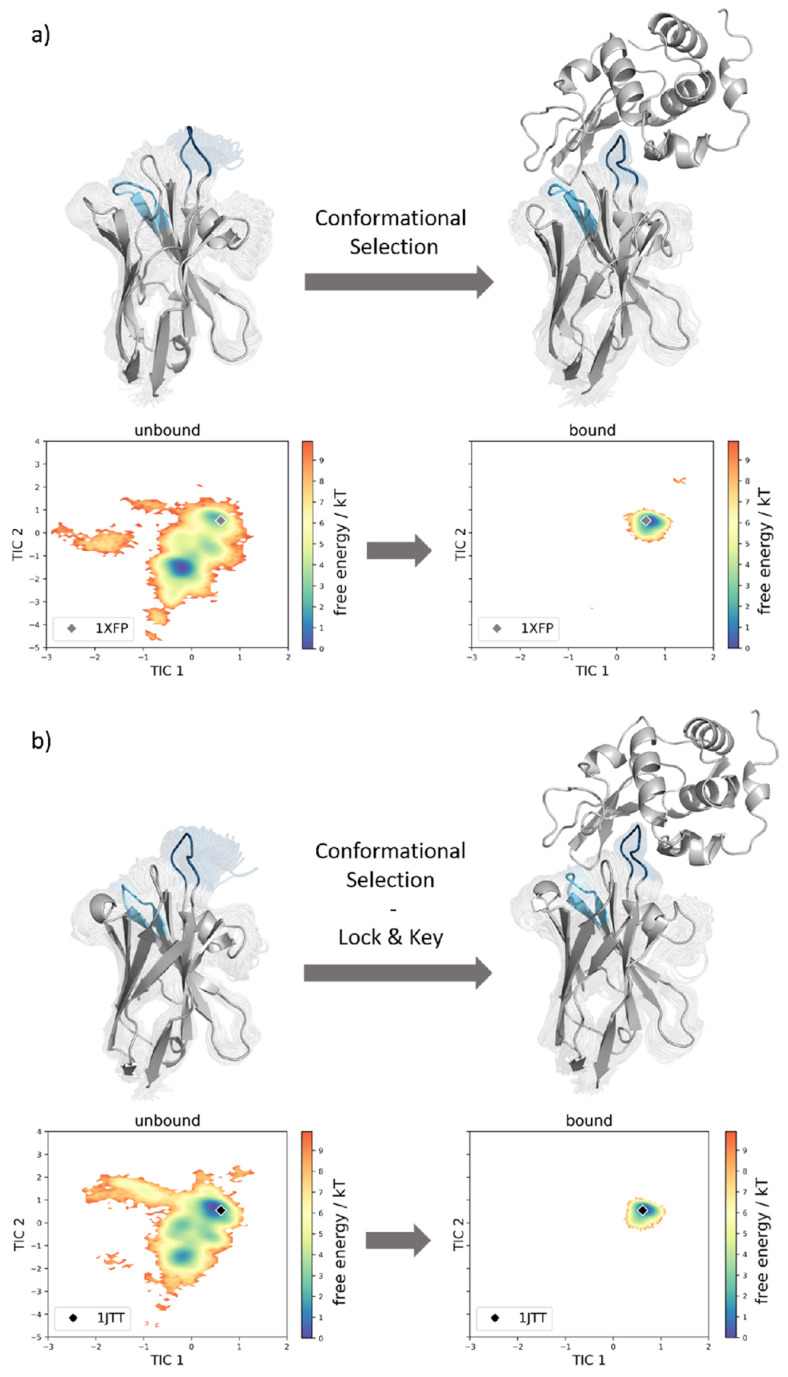
(**a**) Comparison of the structural ensembles resulting from 64.2 μs and 29.0 μs cMD simulation trajectories by simulating the structure with the PDB accession code 1XFP with and without the antigen present. By clustering on the 3 CDR loop, we obtained 321 and 145 clusters, respectively. Next to the structures, the conformational tICA spaces are shown, from which a substantial rigidification is noticeable. (**b**) Comparison of the structural ensembles resulting from 48.0 μs and 35.8 μs cMD simulation trajectories by simulating the structure having PDB accession code 1JTT without and with antigen present. From the clustering on the 3 CDR loops the number of clusters resulted in 240 and 179 clusters, respectively. Next to the structures, the conformational tICA spaces are shown, from which a substantial rigidification is noticeable.

This statement can be improved even more by examining the coordinate systems in Figure 6. Here, the conformational ensembles of the cAb-gl2 variant with and without the presence of the antigen are shown (a), as well as the ensembles of the cAb variant with and without antigens (b). Both of the simulations performed with the antigen present, result in a reduced conformational space. Additionally, we find that the dominant minimum in solution is shifted upon antigen binding for the case of the cAb-gl2, and thus, we suggest that the structure follows the conformation-selection binding mechanism.

For the cAb variant (PDB accession code 1JTT), the dominant minimum in solution in the tICA space of the unbound simulation corresponds to the crystal structure in the complex with the HEL. Therefore, a “lock and key” binding mechanism is suggested. The binding conformation is present also in absence of the antigen beneath many other conformations, but only with the binding partner present, the conformation capable of binding is “locked” and no other conformations are detectable (Figure 6b).

The conformational spaces are mainly characterized by the movements of the binding region of the CDR 3 loop. This can be clarified when looking at the tICA spaces of the loops individually. The major reduction in the extent can clearly be observed for the binding region of the CDR 3 loop (Supplementary Figure S6).

In order to structurally characterize the different V_H_H variants, the interactions of the structures in complex with the HEL were calculated using the GetContacts tool [56]. The results were then depicted as flare plots by use of an in-house python script. As the mutated residues mainly include OH-groups, all inter- and intramolecular hydrogen bond interactions were calculated. From the flare plots, it is visible which residues interact with each other, and also the frequency of these interactions is visible: with the thickness of the lines, the occurrence of the contact over the simulation time increases. Furthermore, the regions which belong to the HEL, and the hypervariable loops are highlighted and color-coded.

**Figure 7:**
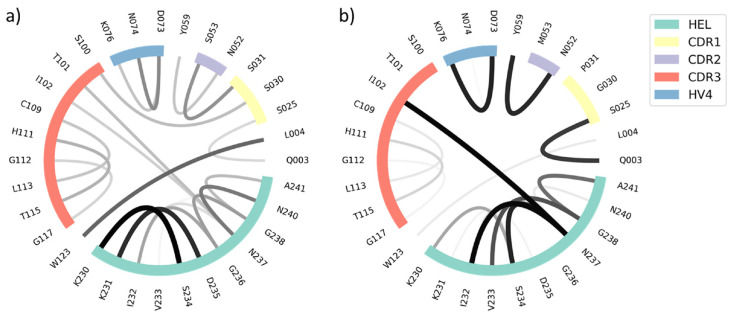
Flare plots showing all inter- and intramolecular hydrogen bond interactions up to the cut-off of 3.5 Å of the (**a**) cAb-gl1+2 and the (**b**) cAb variants. The residues which are part of the CDR loop regions are highlighted according to the colors of the legend. The frequencies of the interactions are represented with the width of the lines. In the variant cAb the number of interacting residues is reduced compared to the cAb-gl1+2, but the intensities are higher.

From Figure 7, it is possible to deduct that the number of interacting residues is minor, but the intensities of the remaining contacts are increased. In particular, one strong contact between the isoleucine in position 102 (CDR 3 loop residue) of the V_H_H domain and asparagine in position 237 of the HEL is found with a much higher probability in the matured variant. Serine 53 was mutated to methionine and therewith the bond to the serine in position 31 of the CDR 1 loop is not present anymore. The stronger and more frequent interloop contacts seem to stabilize the protein, making it entropically favored compared to the variant with lower binding affinity.

## 4. Discussion

During the last decades, the development of antibodies and other biologics as therapeutics has experienced a rapid upswing. Still, many problems are encountered with the handling of such large proteins, which results in the contemporary development of novel and more exotic antibody formats, which are easier to handle. The list of those formats is topped by the smallest alternatives, namely single-domain antibodies comprising only one domain [60,61]. In contrast to conventional IgG antibodies, shark or camelid heavy chain antibodies lack the interface resulting from the pairing of a heavy chain with a light chain. Since the hydrophobic interactions between heavy and light chains are missing, the risk of aggregations is reduced [11,62]. With a size of about 15 kDa, it is even possible for some to pass through the blood–brain barrier (BBB). Thus, V_H_H domains can be applied as carrier molecules as well [63].

The pharmaceutical industry is driven to further improve binding affinities, stabilities and other biophysical properties to optimize antibody–antigen binding and to reduce the risk of developability issues [61,64]. Therefore, it is important to understand and properly characterize structure–function relationships, which help to elucidate the antigen-binding process.

For this purpose, we investigate the conformational diversity of a dataset composed of different V_H_H domains. We chose these single-domain antibodies as two experimentally determined structures were available and the respective dissociation constants have been reported.

To capture a broad conformational space and to overcome high-energy barriers between distinct conformational states, we employed metadynamics as the enhanced sampling technique. Previous studies already discussed the prominent role of the CDR loops in driving antigen recognition [65]. Furthermore, the CDR loops are characterized by their high flexibility. Due to the correlated movements of these loops, single-point mutations in the neighboring loop regions can substantially influence the captured dynamics [66,67,68,69]. It has also been shown how conformational entropy can contribute to the improvement of biophysical properties: stabilizing interactions between loop residues can force the antibody in conformations capable of antigen recognition and binding and they can increase binding affinities. These rigidifications can also decrease the affinity, and with conformational entropy, they have to be considered and optimized in the development process of therapeutic antibodies [70].

In particular, special attention has to be paid to the CDR 3 loop, since this loop is known to play a major role in antigen recognition in conventional antibodies, as well as in T-cell receptors [14,22,71,72]. For the present dataset, conformational spaces have been shown to be determined mainly by the movements of the binding region of the CDR 3 loop.

Dynamics are fundamental for protein recognition, especially the diverse conformational loop ensembles are known to determine antigen binding [14]. It has been previously reported, how protein–ligand binding is mediated by conformational entropy [73]. With the present study, we show the need of taking care of local and global changes in flexibility, but also to consider the respective population probabilities of the obtained conformational ensembles.

Transition timescales and state probabilities of conformational ensembles can directly reflect the chances to allow the antibody–antigen binding process. Furthermore, dynamics can be constrained or also increased because of contacts which contribute to the antibody–antigen recognition and binding process. In the present study, all these factors have been taken into account.

Even though the global flexibility does not change substantially for the analyzed antibody domains, we find a significant population shift of the dominant solution structures in both the PCA and tICA spaces as a consequence of the decrease in the dissociation constant.

The free-energy surfaces have a similar extent, but the deepness of the free-energy surface varies drastically considering the fact that only five residues were mutated within the investigated nanobodies, which consist of approximately 130 residues.

To assess local flexibility, we calculated the root mean square fluctuations (Supplementary Figures S1 and S2). The highest flexibility could be identified for the CDR loop regions, as well as for the HV 4 loop. Astonishingly, with a decrease in the dissociation constants, we find a rigidification of the HV 4 loop (Supplementary Figure S1), even though it is not directly involved in antigen binding, as it is a neighboring loop of the paratope. The HV 4 loop has recently been discussed to play an important role in shaping the antigen binding site and in contributing to antigen recognition [58,74]. This loop can be responsible for stabilizing the binding competent conformation and therefore reducing the conformational diversity of the CDR loops.

By investigating the free-energy surfaces more in detail, we observed that the binding competent conformation was shown to be the most probable structure in the simulation of the variant having the highest binding affinity. Despite this, the binding conformation was also observed in all other simulations, although with lower state probability. This suggests that conformational selection is the binding mechanism on the considered timescale.

The impact of the mutated residues was analyzed: all removed sidechains comprise hydroxy groups, leading to the assumption that hydrogen bonds which originally existed are removed and giving the antibody the opportunity to move towards the binding conformation. Therefore, the deletion of hydrophilic residues could contribute to the recognition of the antigen. The frequency of the contacts increases upon affinity maturation, but the number of different interactions decreases. This indicates an enthalpically favored structure thanks to the introduced mutations, which increase the strength of selected interactions rigidifying the protein in binding competent conformations.

## 5. Conclusions

In this work, we investigated the evolution of maturation of a germline camelid V_H_H domain, including the examination of two intermediate structures. A major objective in the development of therapeutic antibodies is the improvement, or at least the maintenance, of the respective binding affinities. Therefore, it is key to carefully introduce point mutations in order to find the best binding properties.

In this study, we could identify the HV 4 loop region of camelid variable domains as crucial for the stabilization of the binding competent conformation, even if this loop is not directly involved in binding. Of the CDR 3 loop, which is particularly long in V_H_H domains, only a small portion is involved in antigen recognition.

We find strong population shifts upon insertion of up to five point mutations which result in the variant with the highest binding affinity in a stabilization of the binding competent state. This can be explained by changes in the intramolecular hydrogen bond network which was highly variable and flexible for the germline antibody, while the matured variant has a decreased number of overall contacts, which are more frequent and therewith stronger. Thus, our result supports the idea, that upon maturation the antibody is optimized to recognize the antigen.

## Figures and Tables

**Table 1:** Number of clusters obtained by clustering the metadynamics trajectories on the CDR loops 1, 2 and 3 and the resulting aggregated simulation time.

Description	Bound	Unbound
	Number of Clusters/Simulation Time (μs)	
cAb-lys3	179/35.8	240/48.0
cAb-lys3-gl2	145/29.0	321/64.2
cAb-lys3-gl1	156/31.2	223/44.6
cAb-lys3-gl1&2	177/35.4	213/42.6

## Data Availability

All data is included in the manuscript and in the supplementary material. Additional information is available upon request.

## Supplementary Materials

The following supporting information can be downloaded at: https://www.mdpi.com/article/10.3390/biom13020380/s1. Figure S1: RMSF plots to compare the flexibility of the different loop regions of bound and unbound variants; Figure S2: RMSF plots to compare the local flexibilities of the different variants individually; Figure S3: PCA analyses using the dihedral torsions of the CDR2 loop and the binding part of the CDR3 loop as input features.; Figure S4: PCA plots using the backbone-torsions of the individual hypervariable loops as input features.; Figure S5: PCA plots using the cartesian coordinates of the individual hypervariable loops as input features.; Figure S6: tICA plots using the backbone-torsions of the individual hypervariable loops as input features.; Figure S7: Contact analysis of the different bound trajectories.
