# Supplementary material for: Structural Characterization of Nanobodies during Germline Maturation
Source: Biomolecules. 2023 Feb 17;13(2):380. doi: 10.3390/biom13020380 (PMC9953242; doi:10.3390/biom13020380)
Supplement: Supplementary file 1 [file biomolecules-13-00380-s001.zip › biomolecules-2132373-supplementary.pdf]

biomolecules

MDPI

## Supplemental information

### Method: Root Mean Square Fluctuation (RMSF)

The simulation trajectories were analyzed regarding their local flexibility, by calculating the root mean square fluctuations (RMSF) residue-wise (Roe and Cheatham III, 2013).

For the bound simulations, the only reasonable difference could be observed in the HV 4 and in the CDR 1 loops. These regions show an increased flexibility for the cAb-lys-gl1+2 and cAb-lys3-gl1 variants compared to the two variants with higher affinity. For the simulations which were set-up without antigen present, the CDR 1 loop residues are more flexible in the cAb-gl1 and cAb-gl1+2 variants. The CDR 3 loop region which is not directly involved in binding is very rigid for the cAb-gl1 variant, while this part seems to be very flexible for the cAb-lys3-gl2. Upon affinity maturation, for the simulations in complex with the antigen, a rigidification could be clearly observed for the HV 4 loop. The behaviour of the HV 4 loop was previously observed for T-cell receptor variable β-chain domains as well (Fernández-Quintero *et al.*, 2020b).

Furthermore, upon antigen-binding, a strong rigidification in the binding region of the CDR 3 loop was observed in all variants, while for the cAb-gl1 and for the cAb-gl1+2 a rigidification could be observed in the CDR 1 loop as well.

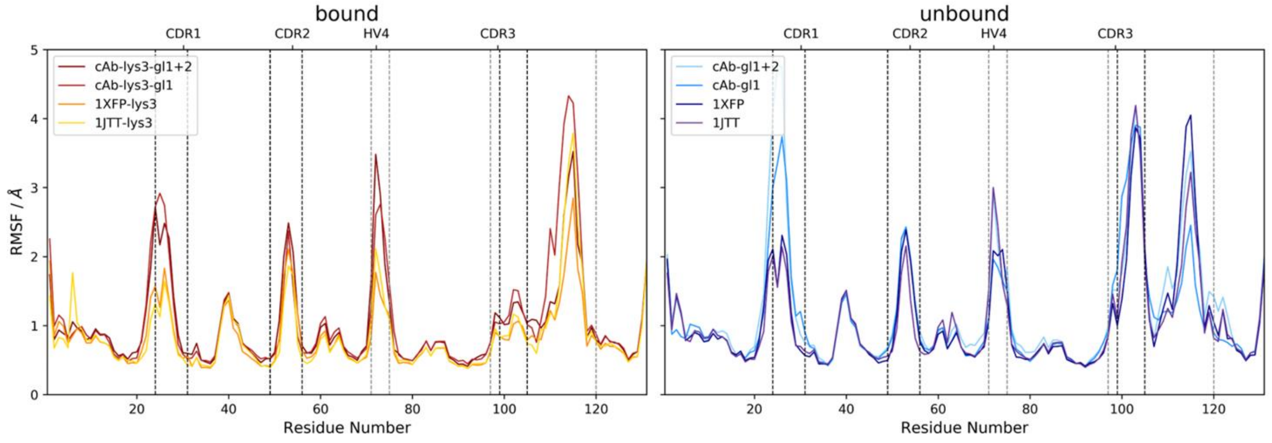


**Figure S1.** RMSF plots to compare the flexibility of the different loop regions of all bound, and unbound variants.

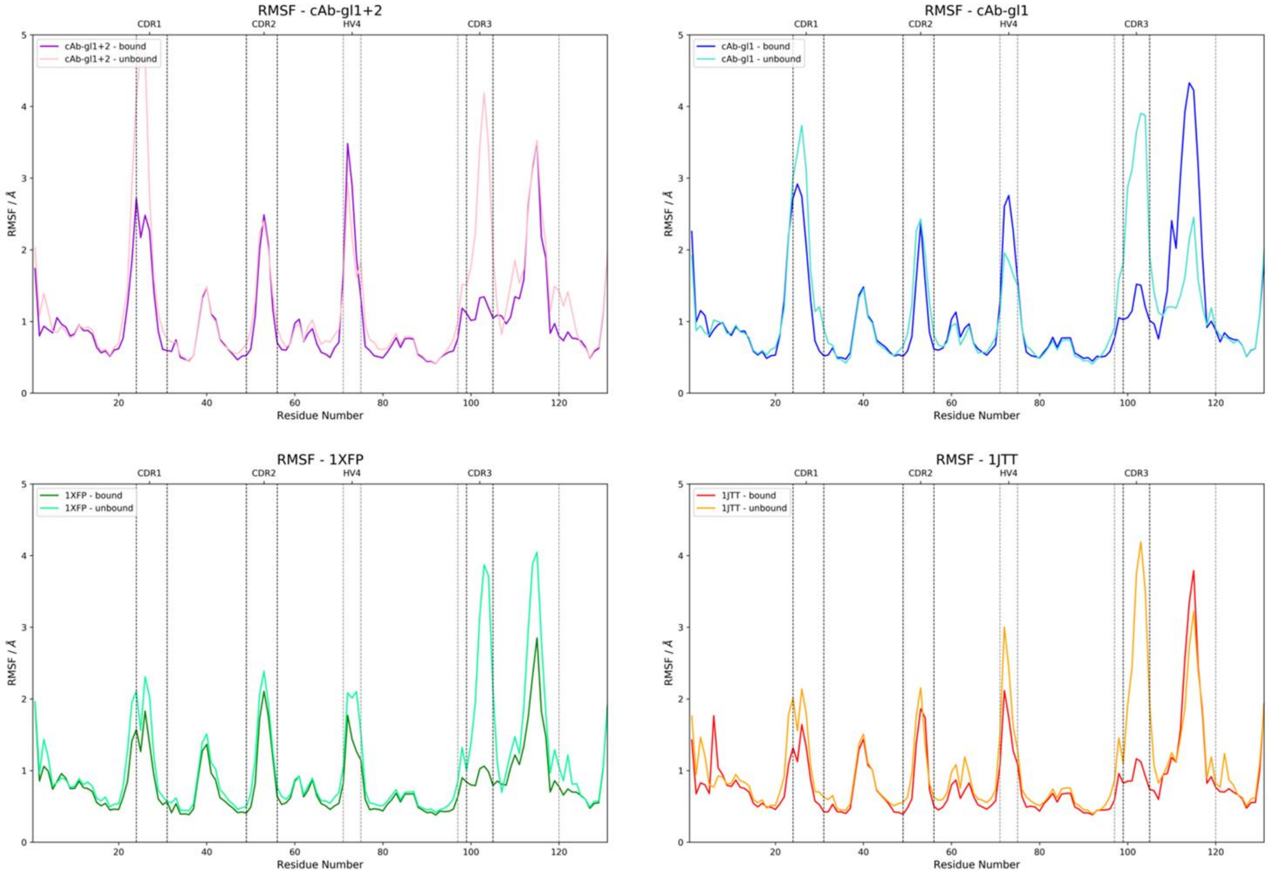


**Figure S2.** RMSF plots to compare the local flexibilities of the different variants individually. For each system, the structure simulated with HEL is compared to the structure simulated without antigen present.

## Method: Principal Component Analysis (PCA)

The simulation trajectories were projected into a combined PCA space to reduce the dimensionality and to be able to directly compare the sampled conformational spaces. Furthermore, the available crystal structures were projected into the same space (Figures S3-S5).

This analysis illustrated in Figures 3 shows that the captured conformational spaces strongly overlap, independently of the binding affinities, but at the same time result in differently populated minima in solution. The binding competent conformation was sampled in all simulations, but with different state probabilities. In particular, the deepest energetic minimum for the binding conformation was encountered for the variant with highest binding affinity.

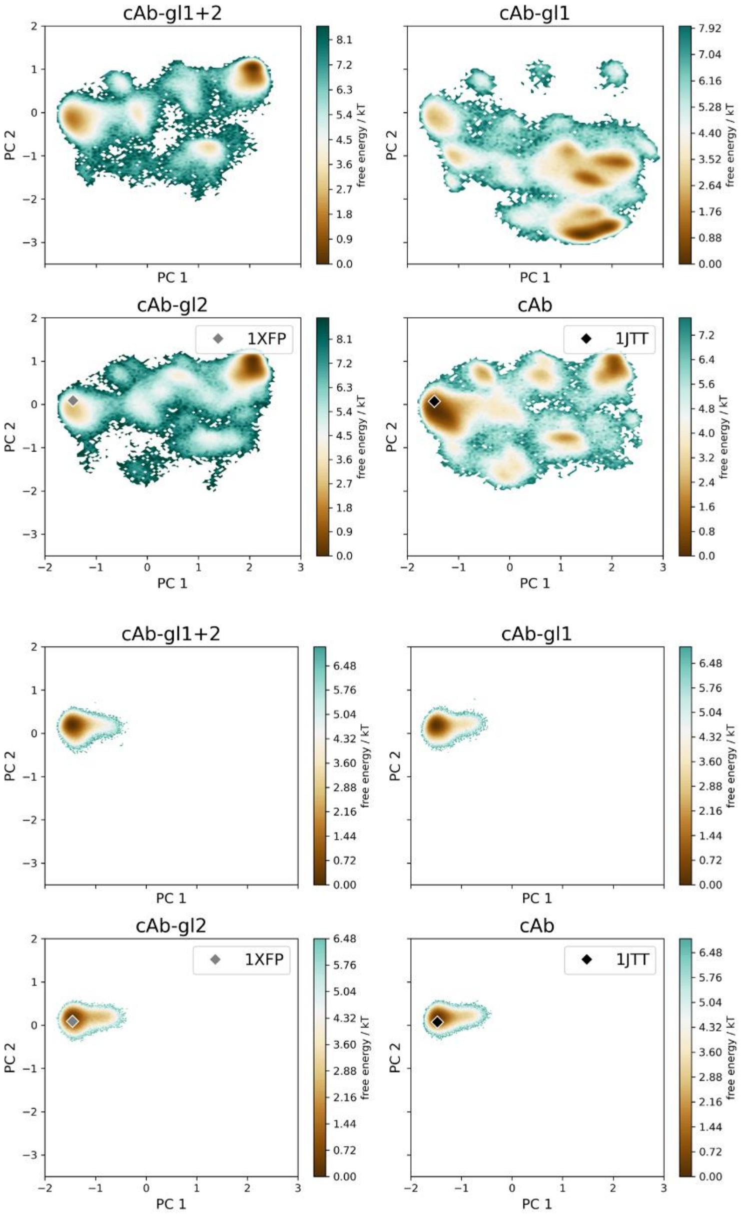


**Figure S3.** PCA analyses using the dihedral torsions of the CDR 2 loop and of the binding part of the CDR 3 loop as input features. The crystal structures of the 1XFP and 1JTT nanobodies are projected in the respective conformational spaces as well.

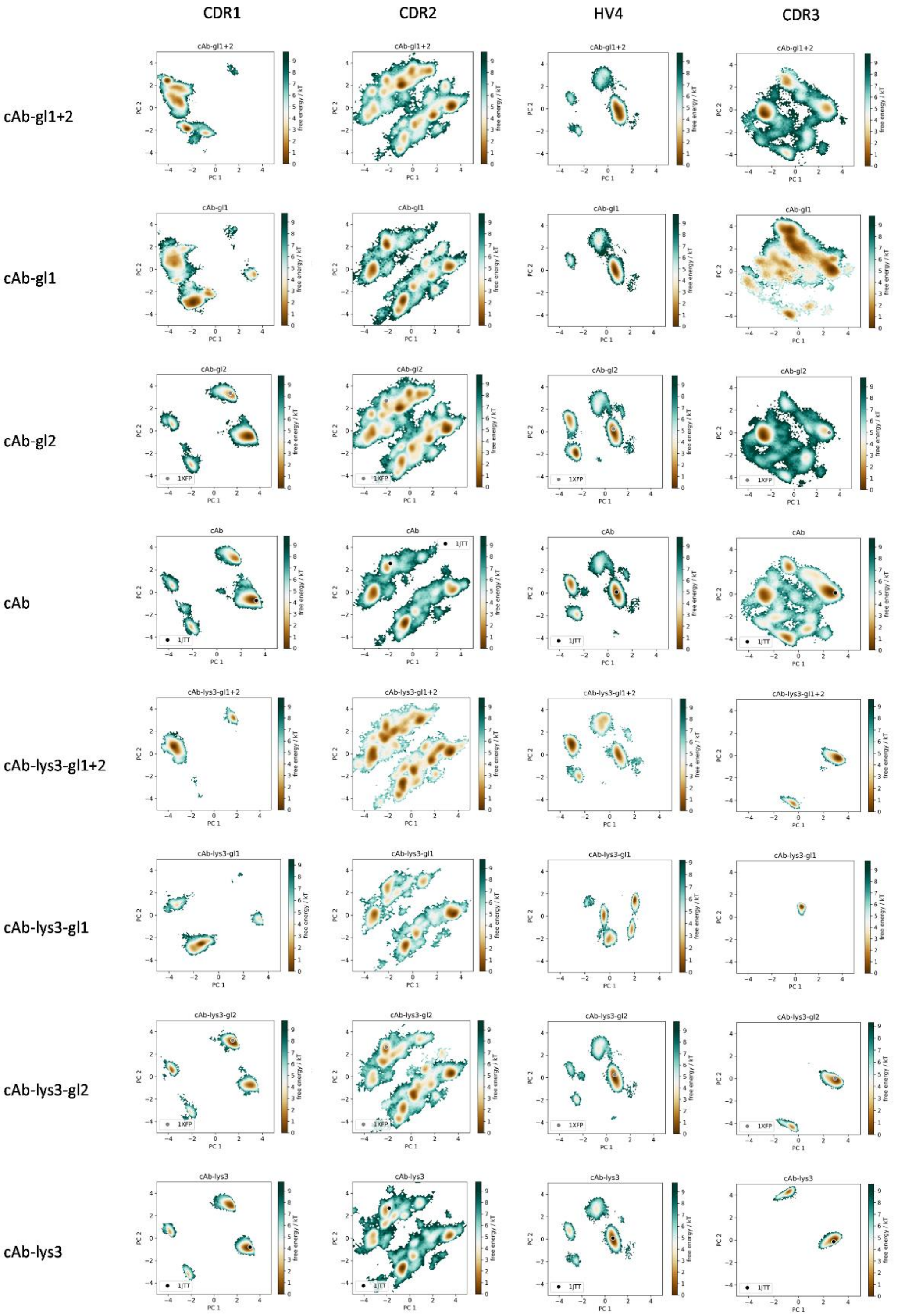


**Figure S4.** PCA plots using the backbone-torsions of the individual hypervariable loops as input features. All conformational spaces were projected into the same coordinate system, and then the different variants shown individually. Where crystal structures are available, these were plotted into the same space as well.

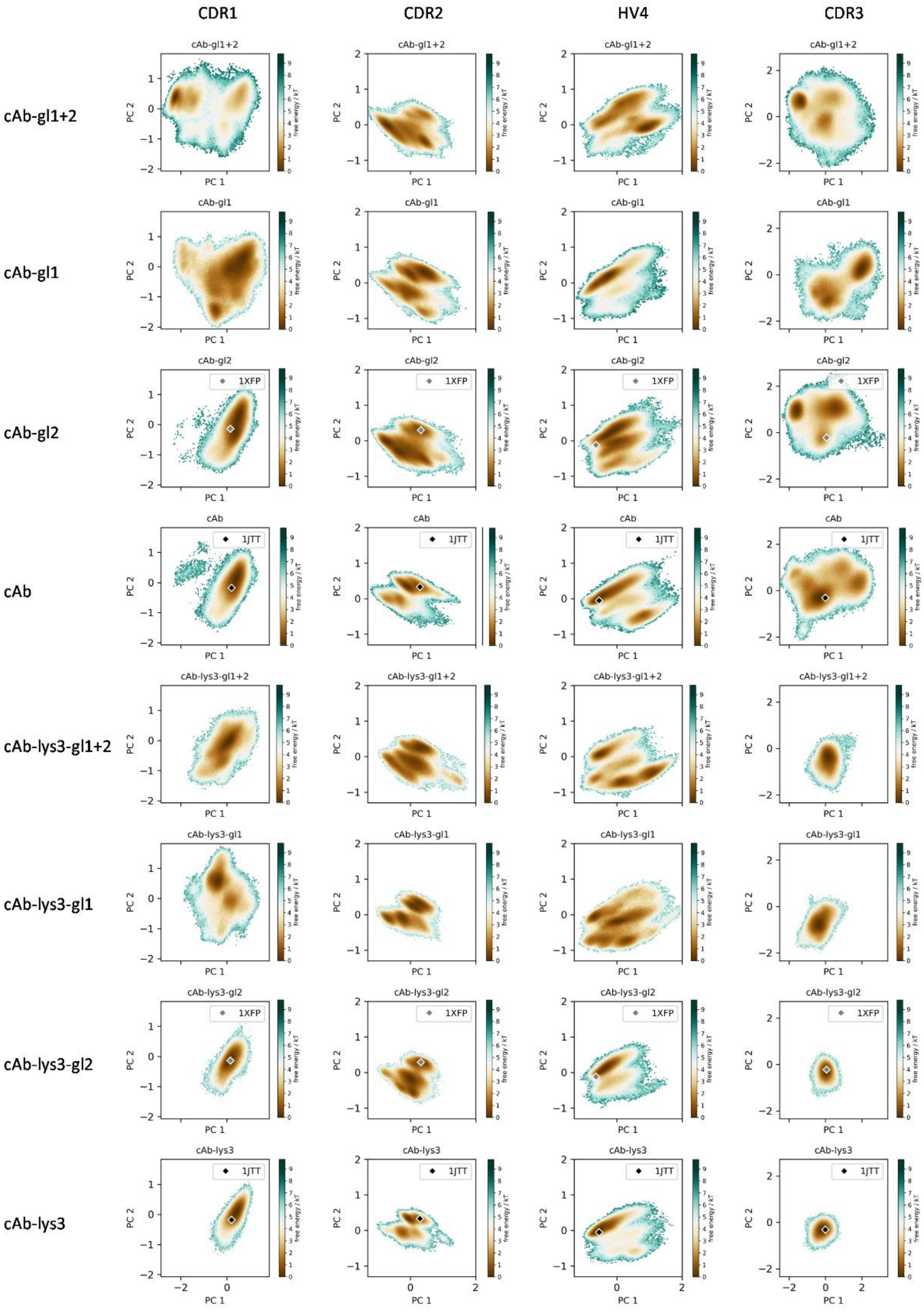


**Figure S5.** PCA plots using the cartesian coordinates of the individual hypervariable loops as input features. All conformational spaces were projected into the same coordinate system, and then the different variants shown individually. Where crystal structures are available, these were plotted into the same space as well.

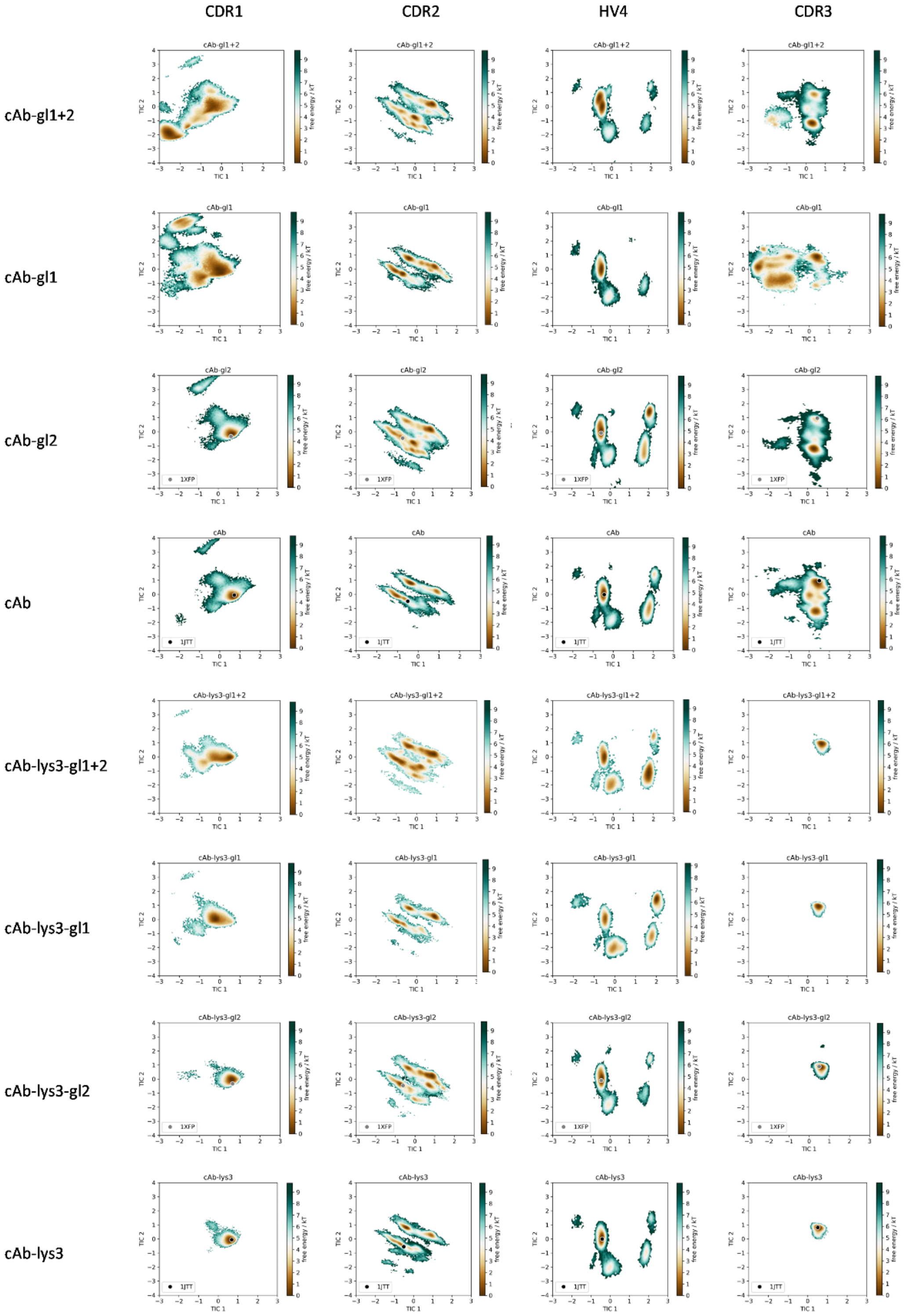


**Figure S6.** tICA plots using the backbone-torsions of the individual hypervariable loops as input features. All conformational spaces were projected into the same coordinate system, and then the different variants shown individually. Where crystal structures are available, these were plotted into the same space as well.

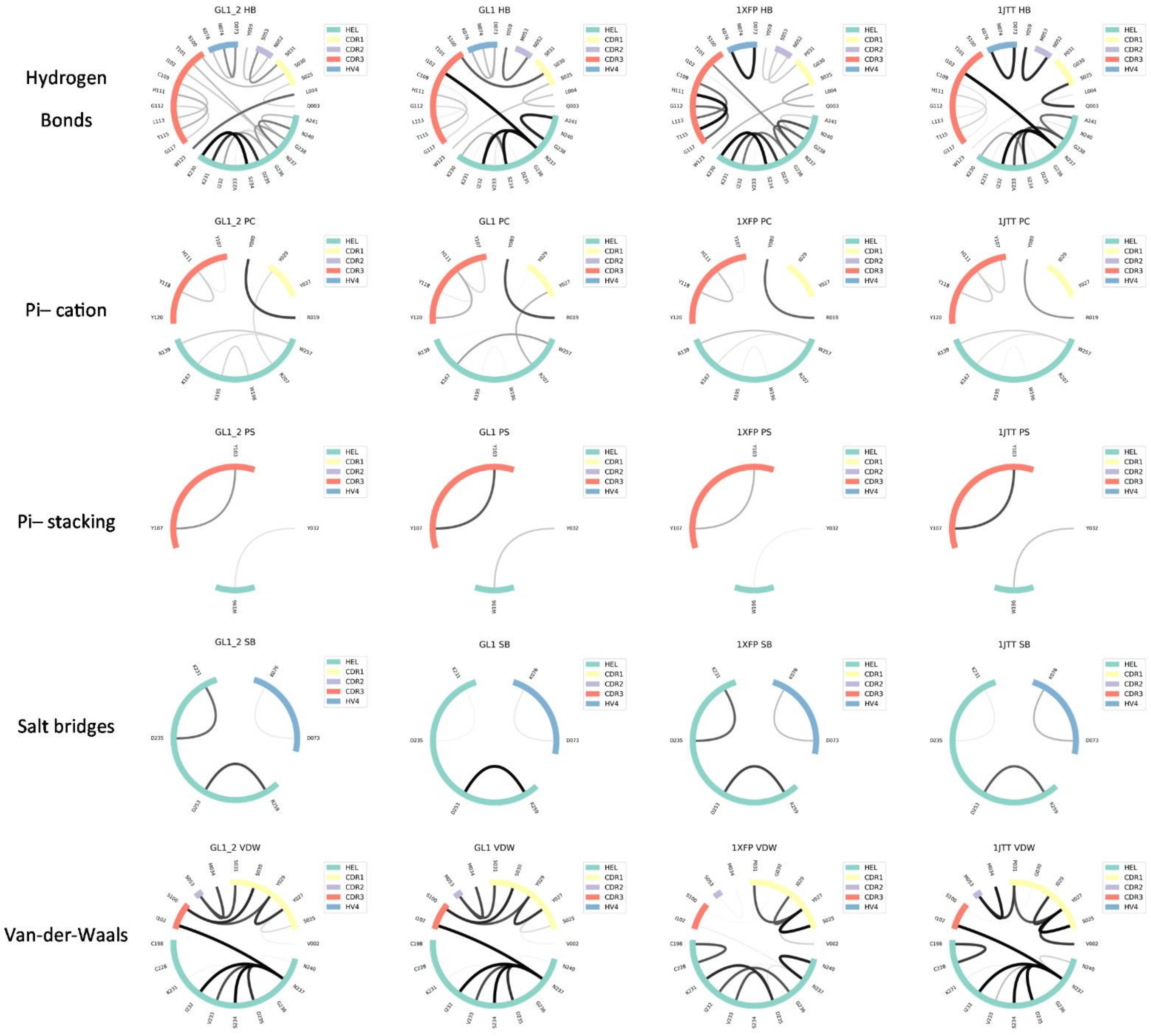


**Figure S7.** Contact analysis of the different bound trajectories. The different interactions types are shown variant-wise. The frequencies of the interactions are represented with the width of the lines.
